# Short-sighted evolution of bacterial opportunistic pathogens with an environmental origin

**DOI:** 10.3389/fmicb.2014.00239

**Published:** 2014-05-20

**Authors:** José L. Martínez

**Affiliations:** Departamento de Biotecnología Microbiana, Centro Nacional de Biotecnología, Consejo Superior de Investigaciones CientíficasMadrid, Spain

**Keywords:** opportunistic pathogen, *Pseudomonas aeruginosa*, cystic fibrosis, chronic infection, bacterial evolution

Evolution is frequently considered as a directional process in which the organisms do not return, after evolving, to a pre-evolved situation. However, there are occassions in which evolution follows a sort of cycling process. Each time an organism is confronted with a given selective situation, it follows a similar evolution path. However, once the selection pressure is resumed, the organism is outcompeted by non-evolved partners and the evolved lineage disappears. This type of process has been dubbed as “short sighted evolution” (Levin and Bull, 1994) and is fundamental for understanding the *in host* adaptation processes of bacterial opportunistic pathogens.

Opportunistic pathogens are a group of microorganisms that do not usually infect healthy hosts but produce infections in hospitals, to immunodepressed persons or those patients presenting underlying diseases as cystic fibrosis, which favors infection (Koch and Hoiby, 1993). Commensal bacteria are among the most prevalent opportunistic pathogens. However, the use of antibiotics, which usually kill commensals besides pathogens, increased the incidence of infections due to environmental microorganisms presenting reduced susceptibility to antibiotics (Bergogne-Berezin et al., 1993).

The evolution of non-pathogenic bacteria towards virulence has been deciphered in occasions. Many “professional” pathogens harbor different sets of genes, dubbed as pathogenicity islands, which have been acquired by horizontal gene transfer, and allow such pathogens to infect the human host (Groisman and Ochman, 1996; Morschhauser et al., 2000; Gal-Mor and Finlay, 2006). The best studied among these evolution processes is that of *Yersinia pestis*, the causal agent of plague (Achtman et al., 2004; Zhou and Yang, 2009). The genus *Yersinia* is formed by 15 different species among which only *Y. pestis*, *Yersinia pseudotuberculosis*, and *Yersinia enterocolitica* are human pathogens. Phylogenetic reconstruction of the evolution of this genus indicates that *Y. pseudotuberculosis* and *Y. enterocolitica* diverged more than 40 million years ago, while *Y. pestis* diverged from *Y. pseudotuberculosis* less than 20,000 years ago (Achtman et al., 1999). The ancestor of these pathogenic *Yersiniae* evolved towards virulence, from a non-pathogenic environmental *Yersiniae*, by acquiring a virulence plasmid named pCD1 (Wren, 2003). This plasmid contains genes coding for a Type III secretion system, which activity allows subversion of the immune system, and is required for the virulence of this pathogen. It is worth mentioning that the acquisition of this plasmid occurred before the divergence between *Y. pseudotuberculosis* and *Y. enterocolitica*, hence more than 40 million years ago; long before the origin of the genus *Homo* (Wood, 1992; McHenry, 1994). This means that the evolution of human pathogens is not exclusively driven by the infection of humans (Martinez, 2013). As stated before, the speciation of *Y. pestis* from *Y. pseudotuberculosis* is a much more recent process that involves both the acquisition of some genes and the loss of other ones. The loss of genes has been frequently associated to genome reduction evolution mostly in the case of endosymbionts (Perez-Brocal et al., 2006). For these microorganisms, several metabolic functions can be covered by the host and un-needed genes are lost just by gene drift. The situation concerning *Yersinia* is not the same. In its process of evolution *Y. pestis* had lost a set of genes encoding insect toxins. Consequently, the evolved organism does not kill insects, a property that allowed its improved transmission by beats of colonized insects (Chouikha and Hinnebusch, 2012). Since this may mean that *Y. pestis* is in the process of evolution toward commensalism in insects, we may conclude that a relevant element for the success of *Y. pestis* as a human pathogen comes from its adaptation to a completely different host, in this case the insects.

As described in this example, evolution towards virulence involves the acquisition of some elements and the loss of other ones. This process leads to speciation from non-virulent towards virulent microorganisms. Whilst this is the situation for several “professional” pathogens, the evolution process of opportunistic bacterial pathogens is rather different. As above stated, these organisms do not infect healthy people and the main factor allowing infection is the patient predisposition for being infected. This may mean that any organism may infect an immunocompromised patient, something that is not fully true.

The human body can be considered as an extreme environment. Actually, when we talk about human microbiota we refer to microorganisms colonizing the surfaces of human body; the body itself is an sterile environment unless an infection occurs. The entrance in this ecosystems requires either the acquisition of virulence determinants to cope with the human defences, as happens with the aforementioned example of *Y. pestis*, either a decay in these defences, as occurs for opportunistic pathogens. However, even in this last case, to colonize the human host a microorganism requires to survive under the physicochemical conditions of human body: a narrow range of temperature oxygen tension and pH; some specific nutrients and low iron availability. In the case of opportunistic pathogens infecting immunocompetent patients as those suffering cystic fibrosis, mechanisms to cope with the immune response are also needed. In addition, and since patients at hospitals are frequently under antibiotic therapy, these opportunistic pathogens frequently display low susceptibility to antibiotics (Martinez and Baquero, 2002). Indeed, antibiotic treatment is a risk factor for being infected by some environmental opportunistic pathogens presenting low susceptibility to antibiotics as *Stenotrophomonas maltophilia* (Alonso and Martinez, 1997; Sanchez et al., 2009).

One of the best studied bacterial opportunistic pathogens is *Pseudomonas aeruginosa*. This microorganism is currently among the most prevalent causes of infection at hospitals and is the major cause of chronic infections in cystic fibrosis patients. These patients are frequently infected by a single *P. aeruginosa* clone that remains and evolves in the lung of the patient for decades (Yang et al., 2011).

Since *P. aeruginosa* is an environmental bacterium, it might be thought that virulent strains constitute a specific branch in the species, which is in its route to speciation as it has happened with “professional” pathogens. Nevertheless, molecular epidemiology has shown that the same clonal complexes present in non-clinical ecosystems are the ones producing infections (Morales et al., 2004; Wiehlmann et al., 2007; Kidd et al., 2012). Further, the comparison of clinical and non-clinical isolates showed that they were functionally equivalent. All of them extruded the synthetic antibiotics quinolones, presented the genes encoding the elements of the Type III secretion system and of the quorum sensing regulation response and were capable to invade epithelial cells; all these characteristics typical of virulent strains. In addition, all strains were able of use oil hydrocarbons as carbon sources, a feature characteristic of environmental biodegradative microorganisms, which is not required for producing an infection (Alonso et al., 1999). Together with the aforementioned epidemiological evidences, this indicates that *P. aeruginosa* does not present two evolutionary branches, one of them towards virulence. Rather, several if not all of the strains have the capability of infecting patients presenting underlying diseases. The reason for this situation resides likely in the ability of *P. aeruginosa* for infecting several different hosts besides humans, including plants, protozoans, worms or insects (Rahme et al., 1995; Tan et al., 1999; Mahajan-Miklos et al., 2000; Navas et al., 2007; Broderick et al., 2008; Carilla-Latorre et al., 2008). Since the same virulence determinants serve to infect all these host it is worth thinking that such determinants evolved during the interaction with the oldest organisms in this phylogenetic tree. In this respect, it is worth mentioning that *P. aeruginosa* is able to avoid being killed by bacteriovorous nematodes and protozoans (Mahajan-Miklos et al., 1999; Cosson et al., 2002). It is then possible that a major force in the evolution of *P. aeruginosa* (and by extension of other opportunistic pathogens with an environmental origin) resides in ancient prey/predator relationships (Martinez, 2013).

All these studies reinforce the idea *P. aeruginosa* did not evolve toward virulence in humans. Rather, this bacterial species can infect humans by using elements that evolved for its interaction with other hosts (Rahme et al., 1995; Mahajan-Miklos et al., 2000). Nevertheless, once a strain enters inside the human host, it can evolve to improve its adaptation to this new environment (Folkesson et al., 2012). Whereas in the case of “professional” pathogens, these changes are usually fixed and contribute to speciation, the same does not necessarily happen in the case of opportunistic pathogens. The evolution of *P. aeruginosa* during chronic infections has been studied in detail. It has been found that strains producing chronic infection carry mutations in global regulation factors that regulate expression of *P. aeruginosa* social cooperative traits, including secretion of exo-products and quorum sensing. As a consequence, expression of these elements, which are of relevance in *P. aeruginosa* acute infections, is reduced in chronic strains (Martinez-Solano et al., 2008; Damkiaer et al., 2013; Jiricny et al., 2014). This in-host evolution process is favored by the increased prevalence of strains presenting high mutation rates found in chronic infections (Oliver et al., 2000; Mena et al., 2008).

It can be thought that these adaptive mutations could be fixed in the population, hence constituting the beginning of a speciation process towards virulence. However, with the exception of some epidemic clones causing infections in cystic fibrosis patients, mainly at Denmark and England (see below), most patients are infected by independent *P. aeruginosa* isolates (Bragonzi et al., 2009), which do not present, during the first stages of infection, the pathoadaptive mutations displayed by the evolved chronic strains. This evolution process by which an organism follows the same pattern of evolution (that is not fixed afterwards) each time it enters in a given ecosystem has been dubbed as short-sighted evolution (Levin and Bull, 1994). This process is likely fundamental for the adaption of microorganisms producing chronic infections with low inter-patient transmission rates.

Although each bacterial isolate colonizes the lung of an independent patient, all lungs are quite similar habitats. It is then expected that the evolutionary landscapes of each of this chronic strains may be similar. However, once bacteria are released, these adaptations are of no use unless the microorganism colonize another cystic fibrosis patient. This is likely the situation of countries as Denmark in which patients have been usually in contact for treatment and in occasions even on holiday camps (Ojeniyi et al., 2000). Similarly, it has been shown that the acquisition of novel prophage islands is a critical event in increasing the competitiveness of epidemic *P. aeruginosa* clones as the Liverpool strain (Winstanley et al., 2009). It could be predicted that under those circumstances epidemic clones, presenting cystic fibrosis adaptive mutations spread, a situation that has been already described in occasions (Jelsbak et al., 2007; Rau et al., 2010; Dettman et al., 2013). Whether or not these epidemic clones are in the route of speciation remains to be established. However, the fact that the implementation of strict cohort-based patients segregation policy has halted the epidemic of the Liverpool strain, with an increase in the percentage of patients with unique *P. aeruginosa* strains (Ashish et al., 2013), indicates this adaptation to be still transient.

In other cases in which inter-host transmission is low, the evolved isolates are released into natural ecosystems. The loss of elements that were fixed, long time ago, during the evolution of *P. aeruginosa* in its natural habitats, make these evolved clones to be outcompeted by the bulk of the population. In this regard, it is worth mentioning that *P. aeruginosa* strains adapted for growing in the lungs of cystic fibrosis patients present a decreased resistance to natural phage and protists predators (Friman et al., 2013).

Short-sighted evolution is a common process in the evolution of microorganisms and has been explained in the frame of a source-sink situation (Sokurenko et al., 2006). In the case of opportunistic environmental pathogens, as *P. aeruginosa*, the majority of the population is present at its original, non-clinical habitat, which constitutes the source of this species. Only a few of these microorganisms infect some patients (sink), just by chance. Evolution during chronic infection allows the adaptation required to persist inside the infected host. However, this happens at the cost of de-adapting for growing in the original habitat, including resistance to natural predators (Friman et al., 2013). The only possibility for the fixation of mutations acquired during chronic infections is a high inter-host transmission, in which case the adapted strain can remain in a habitat (the lung of a different host) that is similar to the one where it evolved without affording the costs of returning to its original non-clinical habitat.

We can then conclude that while the acquisition and loss of genes are at the roots of the speciation of “professional” pathogens, speciation toward virulence is not necessarily required for bacterial opportunistic pathogens. This does not mean however these pathogens do not evolve for a better adaptation for growing in the human host. Indeed, the study of *P. aeruginosa* shows that there are common evolution patterns among isolates producing chronic infections. Nevertheless, these mutations are rarely fixed (with the exception of some epidemic clones Yang et al., 2011, see above); those strains infecting for the first time a patient present in most cases the characteristics of a wild-type strain, not of the ones that evolved during chronic infections. This process that has been dubbed as short-sighted evolution because the organism cannot “foresee” the consequences of such evolution is fundamental for the adaptation of environmental bacterial opportunistic pathogens to the human host.

## Conflict of interest statement

The author declares that the research was conducted in the absence of any commercial or financial relationships that could be construed as a potential conflict of interest.
